# Unmet Need for Family Planning, Contraceptive Failure, and Unintended Pregnancy among HIV-Infected and HIV-Uninfected Women in Zimbabwe

**DOI:** 10.1371/journal.pone.0105320

**Published:** 2014-08-21

**Authors:** Sandra I. McCoy, Raluca Buzdugan, Lauren J. Ralph, Angela Mushavi, Agnes Mahomva, Anna Hakobyan, Constancia Watadzaushe, Jeffrey Dirawo, Frances M. Cowan, Nancy S. Padian

**Affiliations:** 1 University of California, Berkeley, California, United States of America; 2 Ministry of Health and Child Care, Harare, Zimbabwe; 3 Elizabeth Glaser Pediatric AIDS Foundation, Harare, Zimbabwe; 4 Children’s Investment Fund Foundation, London, United Kingdom; 5 Centre for Sexual Health and HIV Research, Harare, Zimbabwe; 6 University College London, London, United Kingdom; University of Washington, United States of America

## Abstract

**Background:**

Prevention of unintended pregnancies among women living with HIV infection is a strategy recommended by the World Health Organization for prevention of mother-to-child transmission of HIV (PMTCT). We assessed pregnancy intentions and contraceptive use among HIV-positive and HIV-negative women with a recent pregnancy in Zimbabwe.

**Methods:**

We analyzed baseline data from the evaluation of Zimbabwe’s Accelerated National PMTCT Program. Eligible women were randomly sampled from the catchment areas of 157 health facilities offering PMTCT services in five provinces. Eligible women were ≥16 years old and mothers of infants (alive or deceased) born 9 to 18 months prior to the interview. Participants were interviewed about their HIV status, intendedness of the birth, and contraceptive use.

**Results:**

Of 8,797 women, the mean age was 26.7 years, 92.8% were married or had a regular sexual partner, and they had an average of 2.7 lifetime births. Overall, 3,090 (35.1%) reported that their births were unintended; of these women, 1,477 (47.8%) and 1,613 (52.2%) were and were not using a contraceptive method prior to learning that they were pregnant, respectively. Twelve percent of women reported that they were HIV-positive at the time of the survey; women who reported that they were HIV-infected were significantly more likely to report that their pregnancy was unintended compared to women who reported that they were HIV-uninfected (44.9% vs. 33.8%, p<0.01). After adjustment for covariates, among women with unintended births, there was no association between self-reported HIV status and lack of contraception use prior to pregnancy.

**Conclusions:**

Unmet need for family planning and contraceptive failure contribute to unintended pregnancies among women in Zimbabwe. Both HIV-infected and HIV-uninfected women reported unintended pregnancies despite intending to avoid or delay pregnancy, highlighting the need for effective contraceptive methods that align with pregnancy intentions.

## Introduction

Although great progress has been made over the past decade in reducing HIV incidence among infants, in 2011, between 280,000 and 390,000 children were newly infected with HIV. [1], [2], [3] The World Health Organization’s (WHO) four-pronged strategy for prevention of mother-to-child HIV transmission (PMTCT) includes reducing the unmet need for family planning among women living with HIV infection, a highly cost-effective HIV prevention strategy. [4], [5], [6], [7], [8], [9] Unmet need is defined as the proportion of women of reproductive age who are married or in a union, are not using contraception, and do not want more children or want to delay the next child. [10] Indeed, family planning is at least as effective as antiretroviral prophylaxis for preventing MTCT and has already averted a large proportion of infant infections. [11], [12], [13] Moreover, it is increasingly recognized that elimination of MTCT will not be possible without further expanding access to contraception among women living with HIV infection. [14] Consequently, eliminating unmet need among all women has been identified as a target towards the elimination of new HIV infections among children by 2015. [15].

Expanding women’s access to and uptake of family planning requires that the available contraceptive methods allow women to optimally align their choice of contraceptive method with their reproductive intentions. For example, although short-acting methods such as oral contraceptives and injectables are the most common in sub-Saharan Africa (SSA), [10] long-acting and permanent methods are more effective under “typical use” conditions, as they eliminate the need for product adherence and have higher continuation rates. [16], [17] However, despite these benefits, long-acting methods are neither widely used nor available in SSA; consequently, contraceptive failure rates could be high even when unmet need is apparently low, given poor adherence to short-acting methods that require daily or quarterly adherence. [18] Access to a variety of contraceptive options is also critical for HIV-infected women who many change their childbearing plans in response to learning their HIV-positive status. [19].

We retrospectively assessed pregnancy intentions and contraceptive use among HIV-positive and HIV-negative women with a recent birth in Zimbabwe, a priority country in the Global Plan towards the elimination of new HIV infections among children by 2015 and keeping their mothers alive. [20] Maternal mortality in Zimbabwe increased at least 28% from 1990 to 2010, [21], [22] with nearly 39% of all maternal deaths being related to HIV/AIDS, warranting further research on women’s access to and demand for family planning. [22] Thus, our objectives were: 1) to understand the frequency of unintended and mistimed pregnancy among women in Zimbabwe; 2) to examine heterogeneity in the frequency of unintended and mistimed pregnancy by HIV serostatus; and 3) to determine, among women with unintended pregnancies, whether HIV serostatus is associated with the use of contraception.

## Methods

We analyzed baseline data from the impact evaluation of Zimbabwe’s Accelerated National PMTCT Program. The accelerated program was initiated in 2011 by the Zimbabwe Ministry of Health and Child Care (MoHCC) and was based on the 2010 WHO PMTCT guidelines. [23] Financial support for the accelerated program was mostly provided by the Children’s Investment Fund Foundation with Elizabeth Glaser Pediatric AIDS Foundation Zimbabwe (EGPAF) as the lead implementing partner. Zimbabwe adopted ‘Option A’ of the 2010 WHO PMTCT guidelines, which recommended that all eligible (i.e., CD4≤350 or WHO clinical stage 3–4) HIV-positive pregnant women receive lifelong ART for their own health, and that HIV-positive women not eligible for ART and their exposed infants receive ARV prophylaxis to prevent MTCT. [23], [24] In addition, ARV prophylaxis was recommended during breastfeeding for all infected women and their children. The impact evaluation will determine the population-level impact of the accelerated program on MTCT and HIV-free child survival at 9–18 months postpartum. [25] The analysis presented here uses data from mother-infant pairs included in the baseline cross-sectional survey of the impact evaluation to examine unmet need for family planning and contraceptive failure.

### Study Population

The target population for the impact evaluation was mother-infant pairs associated with a recent birth. Mother-infant pairs were recruited when infants were 9 to 18 months of age in order to capture MTCT occurring during pregnancy, delivery and during the breastfeeding period. The focus on 9–18 month old infants also permitted collection of baseline data for the impact evaluation, as these infants were born *prior* to the accelerated PMTCT program’s implementation. Women were eligible for inclusion in the impact evaluation (n = 9,018) if they were ≥16 years old and biological mothers or caregivers of eligible infants (alive or deceased). For this analysis, we restricted the sample to 8,662 biological mothers and their eligible infants (alive or deceased) by excluding 356 (3.9%) of 9,018 caregiver/infant pairs.

### Sampling Strategy

The survey was conducted between April and September 2012 in five of Zimbabwe’s ten provinces (Harare, Mashonaland West, Mashonaland Central, Manicaland, and Matabeleland South). These regions were purposefully selected to include Zimbabwe’s capital, rural communities with high and low HIV prevalence, representation of both major ethnic groups in Zimbabwe (i.e., Shona, Ndebele), and areas where detailed monitoring and evaluation data were being collected by EGPAF.

We used a two-stage sampling process. Firstly, we randomly selected 157 catchment areas from 699 health facilities offering PMTCT services in the five provinces, proportionate to the number of facilities in each district. Secondly, all eligible infants were identified and a known proportion per catchment area was sampled, depending on the catchment area’s size (ranging from all infants selected in catchment areas with <150 eligible infants, to 25% of infants in areas with >300 eligible infants). In the case of women with twins or multiple eligible infants, only one infant was selected and referred to throughout the survey.

Potentially eligible infants and their mothers/caregivers were identified by pooling information from: 1) community health workers (CHWs) – individuals who act as liaisons between health facilities and communities, 2) immunization registers from the selected health facilities and their neighboring facilities (to identify women who may access services at adjacent facilities), and 3) mothers identified using CHWs and immunization registers who referred other eligible infants in their neighborhood. Together, this approach efficiently identified eligible participants without screening all households in the selected catchment areas. For example, most Zimbabwean infants receive at least basic immunizations (92–99% [26]), suggesting the reliability of immunization registers. This strategy also captured mother-infant pairs who did not utilize any health services and those who may have accessed care outside of their area of residence (i.e., 35% of mothers enrolled in the survey accessed antenatal care at a different facility than the one corresponding to their place of residence).

### Data Collection

Surveyors visited households with potentially eligible infants and, after confirming their eligibility using a screening questionnaire, a pre-determined sampling fraction (described above) of mothers/caregivers of eligible infants were invited to participate in the study. Mothers providing written informed consent completed an anonymous interviewer-administered survey, including the mother’s characteristics, pregnancy intentions, contraceptive use, and reported HIV status.

### Assessment of Birth Intendedness and Contraceptive Use

To measure intendedness, women were asked “*in terms of the timing of the pregnancy with your baby*” whether she: 1) wanted the pregnancy at that time or sooner (intended), 2) wanted the pregnancy later (mistimed), or 3) was not planning the pregnancy at all (unwanted). [27], [28] Consistent with previous studies, we also included a combined category of unintended pregnancy, which included both mistimed and unwanted pregnancies, irrespective of whether contraception was being used. [28], [29] Women were also asked whether they were using “*any method to prevent or avoid pregnancy*” before they found out they were pregnant; however, the specific contraceptive method type was not assessed. Three mothers (0.03%) with missing data on birth intendedness were excluded from the analysis.

We distinguished between women that presumably experienced a contraceptive failure (i.e., reported that their pregnancy was unwanted or mistimed and that they were using contraception prior to the pregnancy) and those who had an unmet need for family planning (i.e., reported that their pregnancy was unwanted or mistimed and that they were not using contraception prior to the pregnancy). Note that this definition of unmet need includes all women, not just those who were married or in union, which is the definition typically used. [10].

### Assessment of HIV Status

Women were asked about their HIV serostatus prior to learning that they were pregnant, during pregnancy and labor and delivery, and in the 9 to 18 month interval between delivery and the survey. We created a 3-level variable to indicate whether women reported that they were: 1) HIV negative or of unknown status, 2) HIV-infected and aware of their status prior to learning about the pregnancy (HIV-infected aware); or 3) HIV-infected but unaware of their status prior to learning about the pregnancy (HIV-infected unaware). This last group of women reported that they learned their HIV positive status after learning that they were pregnant. We also examined a binary variable of HIV negative or unknown status versus HIV-positive.

### Covariates

We hypothesized that several covariates, which are likely to have temporally preceded the pregnancy and birth, might also be associated with HIV status, birth intendedness, and/or contraceptive use. They were therefore considered for inclusion in multivariable models as potential confounders: province, mother’s age (years), marital status, whether the woman has a regular sexual partner, highest educational level of the woman and male partner (if applicable), ethnicity, religion, household size, whether the mother or mother-in-law lives in the household, the building materials of the best building on the homestead, and lifetime births (including stillbirths). In addition, we created a household asset index using a principal component analysis (PCA) with a polychoric correlation matrix based on ownership of the following household assets: electricity, refrigerator, stove, drinking tap water in the house, livestock, bicycle, motorcycle, car/truck, scotch cart, wheelbarrow, telephone, radio, and television. [30] PCA is a multivariable statistical technique used to reduce a set of correlated variables into fewer dimensions, and avoids the assumptions inherent with a simple summation of assets, namely that all assets are of equal value. [31], [32] The first principal component was used as the asset index, [31] which was divided into quartiles for the analysis. We also created two control variables to indicate whether the infant was alive and the infant’s age in months at the time of the survey (or age the infant would have been, if deceased), indicative of the elapsed time between the pregnancy and the interview. These variables were included to account for potential reporting and recall biases. No more than 1% of any covariate was missing.

### Statistical Analysis

We first performed basic descriptive analyses, including a comparison of baseline characteristics stratified by intendedness of the birth. We then examined intendedness of the birth and contraception use by reported HIV status at the time of the pregnancy.

To determine the association between HIV status and lack of contraceptive use (to understand whether specific groups should be preferentially targeted for future family planning interventions), we constructed a Poisson regression model with HIV status as the exposure and lack of contraceptive use before pregnancy (yes/no) as the outcome, and limited the analysis to women who reported unintended births (unwanted and mistimed). In this model, with cross-sectional data, the exponentiated parameter estimates represent prevalence ratios (PR), a conservative and more interpretable measure of association (compared to the odds ratio) given the frequency of the outcome (47.8% of women with an unintended pregnancy were not using contraception). [33], [34], [35] We ran the model with both a 3-level and binary categorization of HIV status; the adjusted model with the binary categorization yielded similar results to the 3-level categorization so we present the model with the 3-level variable only.

To construct the adjusted regression model, for each covariate, we assessed the presence of a statistical interaction with HIV status to determine whether any interaction terms should be added to the multivariable model. The fully adjusted model contains all covariates specified *a priori* for inclusion (e.g., age), those that changed the log effect estimate by 10% or more (confounders), and those that were strongly associated with the outcome. We checked for multicollinearity between HIV status and other covariates in the fully adjusted model; indicators with variance inflation factors >10 were excluded from models. [36] We present PRs and 95% confidence intervals (CI) computed with linearized standard errors to account for the sample design. The data were analyzed in STATA 12 (College Station, Texas) and were weighted to account for the varying sampling fraction by catchment area and 1.1% survey non-response.

### Human Subjects Protection

This study was approved by the Medical Research Council of Zimbabwe and the ethical review boards at the University of California, Berkeley and the University College London.

## Results

### Participant Characteristics

The weighted population size was 8,797 mothers of infants who were or would have been between 9 and 18 months old at the time of the survey (based on 8,659 observations). Overall, 99.3% of infants were alive with an average age of 14 months. The average age of women was 26.7 years, 92.8% were married or had a regular sexual partner, and they had an average of 2.7 lifetime births (**Table 1**).

**Table 1:** Sociodemographic characteristics of study participants, Zimbabwe, 2012.

Characteristic	Total (N = 8,797)		Birth Intention^b^			
			Intended (N = 5,707)		Unwanted/Mistimed (n = 3,090)	
	N	(%)	N	(%)	N	(%)
Province						
Harare	1,536	(17.5)	1,061	(18.6)	475	(15.4)
Manicaland	3,564	(40.5)	2,344	(41.1)	1,219	(39.5)
Mashonaland Central	1,504	(17.1)	970	(17.0)	534	(17.3)
Mashonaland West	1,338	(15.2)	858	(15.0)	480	(15.5)
Matabeleland South	856	(9.7)	474	(8.3)	382	(12.4)
Age, years (mean, SE)	26.7 (0.09)		26.8 (0.10)		26.6 (0.15)	
Marital status						
Married	7,922	(90.1)	5,324	(93.4)	2,598	(84.1)
Divorced, separated, or widowed	563	(6.4)	253	(4.4)	309	(10.0)
Never married	306	(3.5)	125	(2.2)	181	(5.9)
Married or has a regular sexual partner	8,156	(92.8)	5,431	(95.2)	2,725	(88.2)
Education, highest completed						
No education	274	(3.1)	189	(3.3)	85	(2.8)
Primary school (Standard 7)	2,457	(27.9)	1,496	(26.2)	961	(31.1)
Some secondary school	2,468	(28.1)	1,545	(27.1)	923	(29.9)
“O” Level or higher (Grade 11)	3,595	(40.9)	2,475	(43.4)	1,121	(36.3)
Ethnicity						
Shona	7,346	(83.5)	4,878	(85.5)	2,469	(79.9)
Ndebele	586	(6.7)	326	(5.7)	260	(8.4)
Kalanga/Other	862	(9.8)	500	(8.8)	362	(11.7)
Household size (mean, SE)	5.2 (0.05)		5.0 (0.05)		5.4 (0.06)	
Lives with mother or mother-in-law	1,563	(17.8)	910	(16.0)	654	(21.2)
Asset Index (quartile)						
1st (lowest)	2,459	(28.0)	1,564	(27.4)	896	(29.0)
2nd	1,628	(18.5)	1,070	(18.8)	558	(18.1)
3rd	2,021	(23.0)	1,259	(22.1)	762	(24.7)
4th (highest)	2,684	(30.5)	1,811	(31.7)	873	(28.3)
Lifetime births (mean, SE)	2.7 (0.04)		2.6 (0.04)		2.8 (0.05)	
Infant alive	8,733	(99.3)	5,665	(99.3)	3,069	(99.3)
Infant’s age, months (mean, SE)^c^	13.7 (0.04)		13.7 (0.05)		13.6 (0.06)	

Of the 8,797 births, 3,090 (35.1%) were reported as unintended, including 808 (9.2%) mistimed births and 2,282 (25.9%) unwanted births. Women who reported that their pregnancy was unintended were significantly different in bivariate analyses from women who reported that their pregnancy was intended. Compared to women with intended births, women with unintended births were less likely to live in Harare (15.4% v. 18.6%), less likely to be married or have a main sexual partner (88.2% vs. 95.2%), less likely to have completed O-Level studies (36.3% v. 43.4%), had larger household sizes (5.4 vs. 5.0 people), were more likely to live with their mother or mother-in-law (21.2% vs. 16.0%), and had more lifetime births (2.8 vs. 2.6 births).

One thousand and fifty-nine (12.0%) women reported being HIV-positive at the time of the survey; of these, 584 (55.1%) were aware of their HIV-positive serostatus before the pregnancy and the remaining 475 (44.9%) learned their HIV-positive status during or after pregnancy.

### Birth Intendedness and HIV status

Overall, 3,549 (40.3%) women were using contraception prior to learning that they were pregnant. Of the 3,090 women who reported that their pregnancy was unintended, 1,477 (47.8%) reported using a contraceptive method prior to pregnancy; these women presumably experienced contraceptive failure (16.8% of all births). The remaining 1,613 (52.2%) women who reported that their pregnancy was unintended reported not using a contraceptive method prior to learning that they were pregnant; these women presumably had an unmet need for family planning (18.3% of all births).

The proportion of women reporting that their pregnancies were unintended was similar between women who reported that they were HIV-positive prior to their pregnancy and those who learned that they were HIV-positive after they learned that they were pregnant (44.1% vs. 45.9%, **Table 2**). However, overall, women who reported that they were HIV-infected were significantly more likely to report that their pregnancy was unintended compared to women who reported that they were HIV-uninfected (44.9% vs. 33.8%, p<0.01).

**Table 2:** Intendedness of the birth and contraceptive use prior to pregnancy among women in Zimbabwe, stratified by self-reported HIV serostatus prior to the pregnancy, 2012.^a^

Characteristic	Overall (N = 8,797)		HIV-negative (N = 7,738)		HIV positive (N = 1059)^b^			
					Aware of status (N = 584)^c^		Unaware of status (N = 475)	
	N	%	N	%	N	%	N	%
Intendedness of the birth								
Intended	5,707	(64.9)	5,124	(66.2)	326	(55.9)	257	(54.1)
Unintended	3,090	(35.1)	2,614	(33.8)	258	(44.1)	218	(45.9)
Unwanted	2,282	(25.9)	1,901	(24.6)	203	(34.8)	178	(37.5)
Mistimed	808	(9.2)	714	(9.2)	55	(9.4)	40	(8.4)
Using contraception before pregnancy	3,549	(40.3)	3,047	(39.4)	299	(51.2)	203	(42.7)
Unintended birth and using contraception before pregnancy	1,477	(16.8)	1,217	(15.7)	149	(25.6)	111	(23.3)

a Weighted counts and proportions presented in the table. Numbers may not sum to column totals due to missing data. Percentages may not add to 100 due to rounding.

b Self-reported HIV-positive at any point before, during, or after delivery of the infant.

c Participant reported that she was aware of her HIV-positive serostatus prior to the pregnancy.

All women were ≥16 years old and biological mothers of infants (alive or deceased) born 9 to 18 months prior to the interview.^a^

### HIV Status and Contraception Use Among Women with Unintended Births

**Figure 1:**
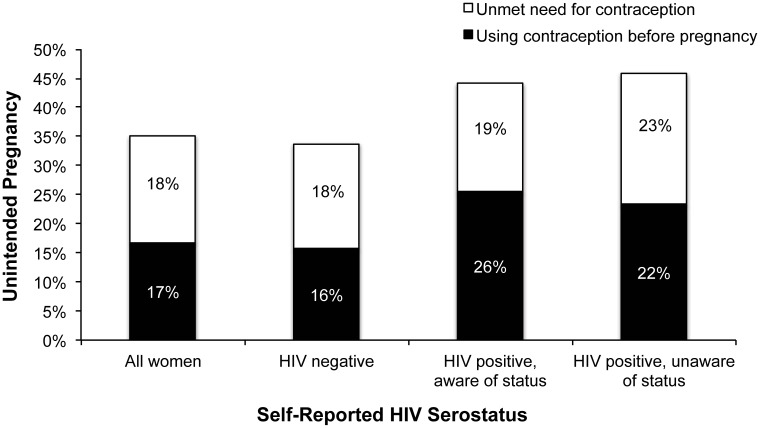
Unintended pregnancy among 8,797 women with a recent birth, stratified by contraceptive use prior to pregnancy and self-reported HIV serostatus, Zimbabwe, 2012. The total column height represents the overall percentage of births that were reported to have been unintended (unwanted or mistimed); the white portion represents the percent of women who were not using contraception prior to the pregnancy (unmet need) and the black portion represents the percent of women who reported using contraception before pregnancy (presumed contraceptive failures)^a^. *a. Percentages of unmet need and contraception use may not sum to column totals due to rounding.*

The prevalence of unintended births is presented in **Figure 1**, stratified by HIV status and contraceptive use prior to pregnancy. Among HIV-positive women who reported that their pregnancy was unintended, there was no difference in pre-pregnancy contraception use for women who did and did not know their HIV-positive status in advance of their pregnancy (58.0% vs. 50.8%, p = 0.23). However, overall, 54.7% of women who reported that they were HIV-positive (regardless of when they learned their status) were using contraception prior to the pregnancy compared to 46.6% of women who reported that they were HIV-negative (p<0.01). After adjustment for covariates, there was no association between self-reported HIV status and lack of contraception use prior to pregnancy (compared to HIV-negative women, HIV-infected aware of their status before the pregnancy: PR_a_ = 0.87, 95% CI: 0.73,1.04; HIV-infected unaware of their status before the pregnancy: PR_a_ = 0.97, 95% CI: 0.82,1.15, **Table 3**).

**Table 3:** Association between HIV serostatus and lack of contraceptive method use before pregnancy among women with unintended births, Zimbabwe, 2012.^a^

HIV serostatus	Unintended birth and not usingcontraception before pregnancy		Unadjusted		Adjusted	
	N	%	PR	95% CI	PR	95% CI
HIV-negative/unknown	1,397	(53.4)	1	–	1	–
HIV positive, aware of status before pregnancy	108	(42.0)	0.79	(0.65, 0.95)	0.87	(0.73, 1.04)
HIV-positive, unaware of status before pregnancy	107	(49.2)	0.92	(0.78, 1.09)	0.97	(0.82, 1.15)

PR: prevalence ratio; CI: confidence interval.

## Discussion

In this study, we found that the unmet need for family planning and contraceptive failure contribute to unintended pregnancies among women in Zimbabwe. Overall, 35% of women with a recent birth in this population-based sample from five provinces reported that their pregnancy was unintended, which is likely a conservative estimate given the potential for underreporting unintended births. [29] Among these women, more than half had an unmet need for family planning and the other half were using a contraceptive method prior to pregnancy and presumably experienced a contraceptive failure. These data highlight the urgent need for women’s access to effective methods that align with pregnancy intentions and that reduce or eliminate the need for daily adherence, including long-acting reversible contraception (LARC). Fortunately, international donors such as the UK Department for International Development (DFID) have recently prioritized increasing the supply of and demand for LARC methods in Zimbabwe. Furthermore, increasing women’s access to and utilization of free comprehensive family planning services is an objective of the Ministry of Health and Child Care and the Zimbabwe National Family Planning Council. [37], [38].

These data are complimentary to the most recent Demographic and Health Survey (DHS), which optimistically found a low unmet need for family planning (13%) and a high prevalence of modern contraceptive use (57%) among women who are married or in union in Zimbabwe. [10], [21] In our study, more than half of the women with an unintended pregnancy had an unmet need for contraception according to our definition – representing 18% of the nearly 8,800 births in the sample. It is not surprising that our estimate of unmet need is different than DHS given that our survey asked women to reflect upon their recent birth, rather than their future fertility preferences. Furthermore, our study includes both unmarried and married women, whereas DHS assesses unmet need only among women who are married or in union, a group whose contraceptive needs and reproductive intentions are likely very different from those of unmarried women. When we limit our analysis to married women, we find that 16% have an unmet need for family planning (data not shown). Thus, after acknowledging the different sample compositions of DHS and our survey, it is valuable to have two large-scale representations of unmet need in Zimbabwe.

The second objective of our study was to examine heterogeneity in the frequency of unintended pregnancy by HIV serostatus. We found that nearly half of HIV-infected women were unaware of their status when they became pregnant, and that the prevalence of unintended births was more than 10 percentage points higher among women who reported that they were HIV-infected at any point in time compared to those who reported that they were HIV-uninfected. Contrary to our expectations, there was little difference in the proportion of unintended births between women who were HIV-infected and aware of their status before they learned that they were pregnant and women who were HIV-infected but learned their serostatus later. This underscores previous reports that for some women, the desire to have children remains strong despite an HIV diagnosis. [19], [39], [40] However, we must nevertheless consider that our study retrospectively assessed pregnancy intention and may be subject to reporting bias, especially if HIV-infected women are differentially counseled to delay or avoid childbearing and over-report unintended pregnancy.

Our final objective was to determine whether HIV serostatus was associated with the lack of contraceptive use prior to pregnancy among women who had an unintended birth. The underlying assumption was that HIV-infected women may have worse access to or demand for contraception than HIV negative women, potentially due to barriers accessing and/or utilizing family planning services or fear of stigma from providers. [41], [42], [43] Overall, we found that approximately half of women with unintended births were actively trying to avoid pregnancy and presumably experienced contraceptive failure. Furthermore, in our adjusted model, there was no association between self-reported HIV status and contraception use, suggesting that, in Zimbabwe, the challenges to obtaining effective contraception apply equally to both HIV-uninfected and HIV-infected women, regardless of when they learned their serostatus. Nevertheless, as increasing numbers of HIV-infected pregnant women are placed on ART for life under PMTCT ‘Option B+’, [44] it will be essential to monitor whether contraceptive availability and the method mix meets the reproductive needs of women living with HIV infection who are also on ART. [45].

Our study analyzed baseline data from a PMTCT impact evaluation that was not specifically designed to examine contraceptive use and pregnancy intentions. Thus, in the absence of prospective contraceptive use data, we assumed that women who reported that their pregnancy was unwanted or mistimed and who reported using contraception prior to the pregnancy experienced a contraceptive failure. In addition, we did not have data about which contraceptive method women were using at the time of their pregnancy, as women were only asked about their current contraceptive method *after* the birth of their infant. Our survey data indicate that 75% of women who were currently using a contraceptive method at the time of the survey, after their baby was born, were using oral contraceptives (data not shown). This is consistent with data from the 2010–2011 DHS which found that that oral contraceptives were the predominant contraceptive method in Zimbabwe, used by 72% of all women who are married or in union and who use any modern method of contraception. [21] Methods that do not require daily adherence are comparatively uncommon in Zimbabwe. For example, among women who are married or in union and who use any modern method, only 14% use injectables and fewer than 5% of women use long-acting methods like intrauterine devices and implants. [21] Thus, although we cannot be certain, it is very likely that the majority of women who potentially experienced a contraceptive failure in our study were using oral contraceptives. Although oral contraceptives are known to have higher typical use failure rates than injectables or long-acting methods, [46] we also must acknowledge the possibility that among HIV-infected women, ART reduces the effectiveness of some types hormonal contraception, an issue that is the ongoing subject of scientific inquiry. [47].

Another limitation of our analysis was that women whose pregnancies ended in miscarriage or abortion were not included in the study. In addition, we assessed HIV status by self-report and our pregnancy intention measure was simple although widely used, including in DHS. Further, our analysis of factors associated with the lack of contraceptive use was cross-sectional and we therefore cannot make inferences about the direction of effect (i.e., temporality) or causation. However, all covariates were purposefully selected to have a high probability of being valid *before* the pregnancy (e.g., marital status, education, religion) and so we do not believe that this is a significant concern. There may also be unmeasured confounders and endogeneity that would bias our estimates of effect, which must be considered when interpreting the results. Finally, deceased mothers are not included in the analysis; these women may be more likely to have been HIV-infected, which would result in our sample of HIV-infected women being under-representative of all HIV-infected mothers. This would also bias our data about intendedness of the birth and contraceptive use. Nevertheless, our analysis has significant strengths, including a large and representative sample and the use of a validated question to determine birth intendedness.

Family planning is important in its own right: it is well-established that modern contraceptive methods prevent unintended pregnancies, reduce maternal and infant morbidity and mortality, and have other significant social and economic benefits. [48] It is also clear that a better understanding of HIV positive women’s fertility intentions and ensuring expansion of family planning services to HIV-infected non-pregnant and pregnant women is key to address WHO’s second prong for PMTCT. [4] Modeling studies have suggested that there will be significant reductions in the number of HIV-exposed infants if unmet need is reduced. [14] In our study, the majority of the HIV-infected women with unintended or mistimed births were actively trying to avoid pregnancy, underscoring the need to expand access to effective family planning in Zimbabwe, including long-acting methods that are less user dependent.
